# Judgments of Learning Reactively Improve Memory by Enhancing Learning Engagement and Inducing Elaborative Processing: Evidence from an EEG Study

**DOI:** 10.3390/jintelligence12040044

**Published:** 2024-04-09

**Authors:** Baike Li, Bernhard Pastötter, Yongen Zhong, Ningxin Su, Ting Huang, Wenbo Zhao, Xiao Hu, Liang Luo, Chunliang Yang

**Affiliations:** 1 School of Psychology, Liaoning Normal University, Dalian 116029, China; baikeli94@gmail.com; 2 Institute of Developmental Psychology, Faculty of Psychology, Beijing Normal University, Beijing 100875, China; 3 Department of Cognitive Psychology and Methodology, Trier University, D-54296 Trier, Germany; pastoetter@uni-trier.de; 4 Collaborative Innovation Center of Assessment for Basic Education Quality, Beijing Normal University, Beijing 100875, China; 5 School of Humanities and Social Sciences, Beijing Institute of Technology, Beijing 100081, China; 6 School of Social Development and Public Policy, Beijing Normal University, Beijing 100875, China; zhaowb@mail.bnu.edu.cn; 7 Beijing Key Laboratory of Applied Experimental Psychology, National Demonstration Center for Experimental Psychology Education, Beijing Normal University, Beijing 100875, China

**Keywords:** judgments of learning, reactivity effect, enhanced learning engagement, elaborative processing, neurocognitive features

## Abstract

Making judgments of learning (JOLs) can reactively alter memory itself, a phenomenon termed the reactivity effect. The current study recorded electroencephalography (EEG) signals during the encoding phase of a word list learning task to explore the neurocognitive features associated with JOL reactivity. The behavioral results show that making JOLs reactively enhances recognition performance. The EEG results reveal that, compared with not making JOLs, making JOLs increases P200 and LPC amplitudes and decreases alpha and beta power. Additionally, the signals of event-related potentials (ERPs) and event-related desynchronizations (ERDs) partially mediate the reactivity effect. These findings support the enhanced learning engagement theory and the elaborative processing explanation to account for the JOL reactivity effect.

## 1. Introduction

Judgment of learning (JOL) is an important form of metacognitive judgment, whereby people predict the likelihood of remembering a studied item in a later memory test (Koriat 1997; Thiede 1999; Thiede and Dunlosky 1999). A large number of studies have found that learners frequently regulate their study activities (e.g., decisions about when, what, and how to study) according to their JOLs. For instance, learners are prone to allocate more time to studying items perceived as less well-studied than to those perceived as well-studied (Dunlosky and Hertzog 1997; Dunlosky and Thiede 2004; Nelson and Narens 1994; Verkoeijen et al. 2005; Yang et al. 2017). These findings reflect that making JOLs can indirectly affect memory through its influence on study activity regulation (Finn 2008; Metcalfe and Finn 2013; Rhodes 2016; Rhodes and Castel 2009). Recent research found that making JOLs can also reactively alter (typically enhance) memory in a direct way, a phenomenon referred to as the reactivity effect (for a review, see Double et al. 2018).

In recent years, many studies have been conducted to explore the reactive influences of making JOLs on memory (Double et al. 2018; Janes et al. 2018; Li et al. 2022; Mitchum et al. 2016; Myers et al. 2020; Rivers et al. 2021; Soderstrom et al. 2015; Tekin and Roediger 2020; Witherby and Tauber 2017; Zhao et al. 2022). However, to the best of our knowledge, no research has yet been conducted to investigate the neurocognitive features associated with the effect. The current study aims to bridge this gap. Below we briefly summarize previous findings related to the reactivity effect, introduce potential mechanisms underlying it, and discuss potential neurocognitive features associated with it.

### 1.1. Reactivity Effect

An emerging set of studies found that making JOLs can reactively change memory itself (Janes et al. 2018; Li et al. 2022, 2023; Mitchum et al. 2016; Myers et al. 2020; Rivers et al. 2021; Soderstrom et al. 2015; Tekin and Roediger 2020; Witherby and Tauber 2017; Zhao et al. 2022). For instance, Li et al. (2022) instructed participants to study four lists of words, with 40 words in each list. For two (JOL) lists, each word was presented for 6 s, and during the last 3 s, participants were required to predict the likelihood of remembering this word in a later recognition test. For the other two (no-JOL) lists, each word also appeared on the screen for 6 s in total, but participants did not need to make JOLs. In a final recognition test, words in the JOL lists were recognized substantially better compared to those in the no-JOL lists, demonstrating a positive reactivity effect (for related findings, see Double and Birney 2019; Yang et al. 2015; Zechmeister and Shaughnessy 1980; Zhao et al. 2022).

Recent studies found that the reactivity effect is long lasting (at least 48 h; Witherby and Tauber 2017) and generalizable to children (Zhao et al. 2022) and young adults (Li et al. 2022), but not to older adults (Tauber and Witherby 2019). In a recent meta-analysis, Double et al. (2018) found a small-to-medium enhancing effect of making JOLs on memory of related word pairs and word lists.

Previous studies mainly employed behavioral methods to explore the JOL reactivity effect (Li et al. 2022, 2023). Going beyond this, the current study incorporates cognitive-neurological techniques (e.g., EEG) to conduct the first exploration of the cognitive neural mechanisms underlying this effect.

### 1.2. Putative Mechanisms

Several theoretical explanations have been proposed to explain why making JOLs reactively affects memory itself. For example, the enhanced learning engagement theory, proposed by W. L. Zhao et al. (2022), hypothesizes that the reactivity effect is derived from enhanced learning engagement induced by the requirement of making JOLs (Tauber and Witherby 2019; Tekin and Roediger 2020; Witherby and Tauber 2017; Zhao et al. 2022). Specifically, participants’ attention gradually wanes, and mind wandering systematically increases across a learning task, leading to weakened learning engagement and inferior learning outcomes (Seli et al. 2016). However, when they are asked to make a JOL for each study item, they have to sustain their attention on the learning task (that is, they have to closely encode and analyze the study items in order to find “diagnostic” cues to provide a reasonable JOL for each item). Therefore, the requirement of making item-by-item JOLs should reduce (or even prevent) attention waning and enhance learning engagement, which in turn produces a positive reactivity effect.

Tauber and Witherby (2019) provided a similar explanation to account for their age-difference findings: that is, making JOLs reliably enhances young adults’ memory, but fails to benefit older adults’ memory. They assumed that older adults, compared with young adults, are typically equipped with greater learning motivation (Jordão et al. 2019), and their minds wander less frequently than young adults’ minds (Einstein and McDaniel 1997; Krawietz et al. 2012). Therefore, making JOLs is less effective in enhancing older adults’ learning engagement than it is for young adults, leading to a smaller or no reactivity effect for older adults (Tauber and Witherby 2019).

Another possible explanation for JOL reactivity is the elaborative processing account, which assumes that making JOLs enhances retention by inducing more elaborative processing (Li et al. 2022; Soderstrom et al. 2015; Tekin and Roediger 2020; Zechmeister and Shaughnessy 1980). Specifically, making item-by-item JOLs may drive participants to adopt more elaborative study strategies to process the study items, which in turn produces a positive reactivity effect (Mitchum et al. 2016; Sahakyan et al. 2004). For instance, Sahakyan et al. (2004) found that asking participants to make a JOL (i.e., predicting the number of words they would remember in a later memory test) following the study of a word list caused them to shift from poor learning strategies (e.g., rote rehearsal) to more effective ones during the subsequent study of a new word list. Tekin and Roediger (2020) found that words receiving shallow processing (e.g., perceptual judgment) exhibited a larger reactivity effect than those receiving deep processing (e.g., semantic judgment). The interaction between reactivity and level of processing suggests that the reactivity effect may result from the fact that making JOLs induces more elaborative processing. Furthermore, it has been shown that making JOLs promotes item-specific processing of study items, in turn producing superior recall or recognition performance (Chang and Brainerd 2024; Senkova and Otani 2021; Zhao et al. 2023a, 2023b).

The enhanced (attentional) learning engagement and elaborative processing explanations may jointly explain the positive reactivity effect of JOLs on memory. For instance, Shi et al. (2023, Experiment 3) recruited participants to learn four lists of pictures. In two lists, participants were asked to make JOLs during learning, whereas in the other two lists, they were not asked to make JOLs. In addition, Shi et al. inserted some probes to detect participants’ mind wandering during the learning phase. After the final test, participants were asked to subjectively report the learning strategies they used during the learning phase. Shi et al. found a positive reactivity effect on visual memory. More importantly, participants reported less frequent mind wandering in the JOL than in the no-JOL condition, and the frequency of mind wandering partially mediated the positive reactivity effect. In addition, among the participants who showed a positive reactivity effect, 41.1% of them reported that they remembered more images in the JOL condition because they used superior learning strategies (e.g., focusing on the visual features of pictures). The study by Shi et al. (2023, Experiment 3) thus suggests that enhanced learning engagement and increased level of elaborative processing may jointly contribute to the JOL reactivity effect.

Although some studies provided suggestive evidence that making JOLs may promote elaboration processing, other studies found that participants used similar learning strategies between the JOL and the no-JOL conditions when they were instructed to subjectively report which study strategies they used after they finished the final test (Rivers et al. 2021; Soderstrom et al. 2015). This may derive from the fact that, after the final test, participants’ subjective reports may confuse their memory contexts between the JOL and no-JOL conditions. To explore whether making JOLs increases the level of elaborative processing, the current study used electroencephalography (EEG) to record electrical activities elicited on the scalp of the brain during the encoding phase. By recording electrical activities elicited on the scalp, the indexes of event-related potentials (ERPs) can provide more evidence for the enhanced learning engagement and elaborative processing explanations.

Before continuing, it is worth noting that there are several other explanations of the reactivity effect, such as the cue-strengthening theory (Rivers et al. 2021; Soderstrom et al. 2015) and the changed goal theory (Janes et al. 2018; Mitchum et al. 2016; Li et al. 2024). Because these theories are not directly related to the current study, we do not discuss them further. Interested readers can consult Mitchum et al. (2016) and Soderstrom et al. (2015).

### 1.3. Cognitive Neural Indicators Associated with the Reactivity Effect

The present study is the first to explore electrophysiological correlates of the JOL reactivity effect. As previously mentioned, behavioral research suggested that both enhanced learning engagement (Tauber and Witherby 2019; Tekin and Roediger 2020; Witherby and Tauber 2017; Zhao et al. 2022) and elaborative processing (Li et al. 2022; Soderstrom et al. 2015; Tekin and Roediger 2020; Zechmeister and Shaughnessy 1980) may contribute to the JOL reactivity effect. Accordingly, regarding the electrophysiological correlates of the reactivity effect, we predicted that components of ERP waveforms related to attentional (e.g., P200) and elaborative processing (e.g., LPC) would be related to the magnitude of the JOL reactivity effect.

P200 is a positive-going waveform component, and its peak varies in latency from 150 to 280 ms following the onset of stimuli. Many studies have established that P200 and P200-like components (e.g., P240) relate to attentional engagement during the encoding phase of episodic memory tasks (Carreiras et al. 2005; Gan et al. 2016; Kanske et al. 2011; Leuthold et al. 2015; Lu et al. 2010; Missonnier et al. 2007). For instance, Gan et al. (2016) found that compared with moral words, immoral words attract more attention and induce a larger frontal P200. Kanske et al. (2011) found that emotional words, compared with neutral words, capture greater attention and induce a larger P200 during a vocabulary learning task. Based on these findings and according to the enhanced learning engagement theory, we predicted that making JOLs would elicit larger P200 amplitudes.

The late positive component (LPC) occurs at 500 to 900 ms post stimulus and is often observed over the whole scalp with a maximum over parietal electrodes. Prior research demonstrated that the parietal LPC correlates with elaborative processing in episodic memory tasks (Beato et al. 2012; Fortin et al. 2021; Gan et al. 2016; Kim et al. 2020; Packard et al. 2020; Sanquist et al. 1980; Zhang et al. 2020). For instance, Sanquist et al. (1980) found that deep processing (e.g., judging whether two words are semantically related) enhanced memory performance and elicited greater LPC amplitudes compared to surface processing (e.g., judging whether two words are different in orthography). Based on these findings and according to the elaborative processing theory, we predicted that making JOLs may induce larger LPC amplitudes during the encoding phase.

The EEG data can also be analyzed concerning local synchronization of neural oscillations, as measured by event-related synchronization (ERS; i.e., power increase) and event-related desynchronization (ERD; i.e., power decrease) within specific frequency bands (e.g., alpha and theta) over single electrodes (Pfurtscheller 1992). Neural oscillations can also reflect the cognitive processes during a learning task (Jia et al. 2021; Katerman et al. 2021; Pastötter et al. 2008; Pastötter et al. 2011; Van Strien et al. 2007). For instance, Pastötter et al. (2011) found that undertaking a practice test after studying each word list, compared with restudying, can effectively enhance participants’ learning engagement across a multiple-list learning task, as reflected by greater desynchronization of alpha power in the test than in the restudy condition (Pastötter et al. 2008; Pastötter et al. 2011). More importantly, Pastötter and colleagues found that alpha desynchronization successfully predicted subsequent memory performance. Based on these findings and according to the enhanced learning engagement theory, we expected to observe that making JOLs would induce greater alpha desynchronization during the encoding phase.

Previous studies have also found that beta (13–30 Hz) desynchronization is correlated with semantic elaboration and deep encoding of item information (Guran et al. 2019; Hanslmayr et al. 2008; Klimesch et al. 1997; Pastötter and Bäuml 2016). EEG-fMRI studies found that beta band desynchronization is associated with increased activation of the left ventrolateral prefrontal cortex, which is involved in elaborative processing of study items (Hanslmayr et al. 2011). If making JOLs induces a positive reactivity effect via increasing level of elaborative processing, we would expect to observe that making JOLs should increase desynchronization of the beta band.

### 1.4. The Current Study

The goals of the current study were to replicate the behavioral reactivity effect and examine its electrophysiological correlates in ERP and time–frequency data. ERPs only measure evoked brain activity. By contrast, ERS and ERD can measure both evoked (i.e., phase-locked) and induced (i.e., not-phase-locked) brain activities (see Tallon-Baudry and Bertrand 1999). In the current study, we subtracted the ERP from single trials in the ERS/ERD analysis, and therefore examined induced activities only with time–frequency analysis. We expected to observe a behavioral reactivity effect. More importantly, according to the enhanced learning engagement and elaborative processing explanations, we expected to observe differences in the components related to attentional engagement (e.g., P200 amplitude, alpha desynchronization) and elaborative processing (e.g., LPC, beta desynchronization).

## 2. Method

### 2.1. Participants

A power analysis was conducted via G*Power (Faul et al. 2007). The power analysis for a one-tailed, paired *t*-test indicated a required minimum sample of 27 participants to find a medium-sized EEG effect when the level of significance was set to 0.05 and power (1-beta) to 0.80. Note that many recent studies observed that the behavioral reactivity effect on recognition memory of word lists is a large-size effect (e.g., Cohen’s *d* = 1.228 in Li et al. 2022) and thus should be well replicable with this sample size (beta < 0.001). Finally, we collected data from 30 participants. Due to the experimental program crashing, data from one participant were unsaved, and another two participants’ data were excluded because of poor quality EEG data.

The final data from 27 participants (*M* age = 21.519, *SD* = 2.765; 8 males) recruited from Beijing Normal University (BNU) were analyzed. All participants were right-handed, were not taking any psychotropic medications, and did not have any history of neurological diseases. They provided written consent and received monetary compensation. The protocol was approved by the Institutional Review Board of BNU’s Faculty of Psychology.

### 2.2. Materials

The stimuli were 620 two-character Chinese words extracted from the Chinese word database developed by Cai and Brysbaert (2010). The word frequency of these words ranged from 2.53 to 50.94 per million. Twenty words were used for practice and the other 600 words were used in the formal experiment. For each participant, 400 words were randomly selected by computer to be presented during the study phase, which also served as “old” items in the recognition test, with the other 200 words serving as “new” items in the recognition test.

To prevent any item selection effects, for each participant, the 400 to-be-studied words were randomly divided into four lists, with 100 words in each list. Two lists were randomly assigned to the JOL condition, and the other two lists were assigned to the no-JOL condition. In addition, the presented sequence of words in each list and the list sequence were randomly computed for each participant. All stimuli were presented via Matlab *Psychtoolbox* (Kleiner et al. 2007).

### 2.3. Procedure

After participants signed an informed consent form, the EEG was prepared. This took about 15~45 min. Participants were comfortably seated about 60 cm from a computer screen. Next, instructions about the learning task were provided on the computer monitor. The EEG was recorded only during the learning session. After the learning session, the EEG cap was removed and an old/new recognition test was implemented. Overall, the experimental session took a maximum of 150 min.

Following previous JOL reactivity studies (Kubik et al. 2022; Li et al. 2023, 2024), the current experiment employed a within-subjects design (JOL vs. no-JOL). Participants were informed that they would study four lists of words in preparation for a later memory test. For two lists, they would be asked to predict the likelihood of remembering each word in a later memory test. For the other two lists, they would press a number key on the keyboard corresponding to a digit presented on the screen. Importantly, they were informed that they needed to remember all words equally well regardless of whether they had to make memory predictions or press a number key in response to the on-screen digit because all words would be finally tested. Before the formal experiment, participants completed a practice task to familiarize themselves with the experimental procedure. The procedure of the practice task was the same as that of the main experiment.

**Figure 1:**
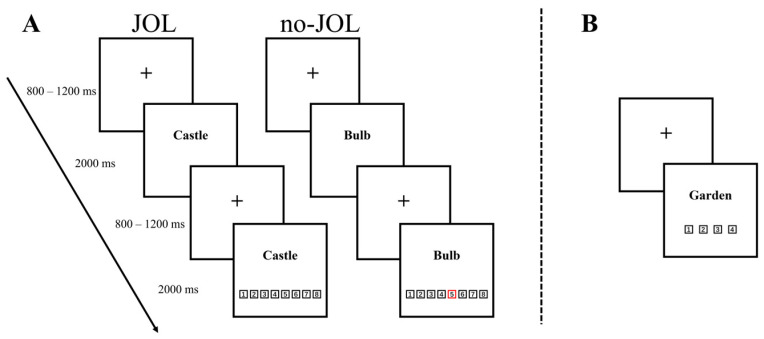
Sequence of study trials (**A**) and test trials (**B**). During the study phase, participants studied a word and then made a JOL (JOL list) or pressed a number key circled by red rectangle (no-JOL list). During the test phase, participants were asked to indicate whether the on-screen word was old or new.

In the formal experiment, participants studied four lists of words, with 100 words in each list. Before studying each list, the computer informed participants whether or not they would need to make memory predictions for the following list of words. As shown in Figure 1, a study trial began with a fixation cross (duration: 800–1200 ms), followed by the first appearance of the word for 2000 ms. After another fixation cross (duration: 800–1200 ms), the word was shown again with 8 digits (1–8) presented below it. In the JOL lists, participants were instructed to predict how likely it was that they would remember the word in a later memory test. Their predictions were made on the scale ranging from 1 (*Sure I will not remember it*) to 8 (*Sure I will remember it*). The scale was presented for 2 s, and participants made their JOLs by pressing the number keys on the keyboard. In the no-JOL lists, one of those digits was randomly selected by the computer and circled by a red frame, participants were instructed to press the number key in response to this digit.

If they successfully made a response within the 2 s time window, the word remained on screen for the remaining duration of the 2 s to ensure that the total exposure time for each word was equal between the JOL and no-JOL conditions. If they did not make a response during the required time window, a message box appeared to remind them to make responses for the following words during the required time window. Participants pressed the “Space” key to remove the message box and start the next trial.

After participants studied all words, they solved math problems (e.g., 7 + 45 = ___?) for 5 min, which served as a distractor task. And after 5 min break, the old/new recognition test began. The 400 studied (old) and 200 new words were presented one by one in random order. Participants were asked to decide whether each word presented on screen was old or new on a four-point scale, with 1 = “*definitely new*” and 4 = “*definitely old.*” The stimulus remained on the screen until a response was made. There was no feedback and no time pressure in the recognition test.

### 2.4. Behavioral Data Analyses

To examine the behavioral reactivity effect (Li et al. 2022, 2023), discriminability (*d′*, an index reflecting the ability to discriminate the signal [i.e., old words] from the noise [i.e., new words])^1^, and response criterion (*c′*, an index reflecting an individual’s propensity for the “old” response in the recognition test) were calculated (for detailed explanations of d′ and c′, see Banks 1970). Old words receiving a response of 3 or 4 in the recognition test were considered as “hits,” and new words receiving a response of 3 or 4 were coded as “false alarms.” Following precedents (e.g., Winograd and Vom Saal 1966; Yang et al. 2015), the main measure of recognition performance employed in the current study was *d′*. The results of item-by-item JOLs are reported in Appendix A, which are not the main research interest.

### 2.5. EEG Recording and Preprocessing

EEG data were recorded from 64 Ag/AgCl electrodes embedded in an elastic cap equipped with a NeuroScan SynAmps system at a sampling rate of 1000 Hz with a 0.05–125 Hz band-pass filter. All electrodes were referenced to an electrode positioned between CPz and Pz. The electrodes M1 and M2 were placed on the left and right mastoids, respectively. All impedances were kept below 5 kΩ.

EEG data were preprocessed with EEGLAB (Delorme and Makeig 2004). The sampling rate was reduced to 500 Hz in offline processing. The offline data were re-referenced to the average of M1 and M2. EEG activity was re-filtered offline from 0.5 Hz to 45 Hz, band-pass, zero-phase shift digital filter. Ocular artifact reduction was performed through ICA component rejection (Jung et al. 1998), and other movement artifacts were identified by visual inspection and manually removed. Peripheral electrodes were excluded from EEG analysis because they showed sustained artefacts in various participants. Therefore, the EEG analysis was restricted to 40 electrodes: F5, F3, F1, FC5, FC3, FC1, F6, F4, F2, FC6, FC4, FC2, Fz, FCz, C5, C3, C1, CP5, CP3, CP1, C6, C4, C2, CP6, CP4, CP2, Cz, CPz, P5, P3, P1, PO5, PO3, P6, P4, P2, PO4, PO2, Pz, and POz.

Continuous recordings were segmented into stimulus-locked epochs ranging from −1000 to 2000 ms around stimulus onset of the first word presentation within a trial. The second word presentation was not analyzed due to confounding influences of the preparation and execution of hand movements.

### 2.6. ERP Data Analyses

ERPs were baseline corrected. The baseline was set from −300 to 0 ms before the onset of stimuli. To control for problems of multiple comparisons when testing the significance of amplitude differences over multiple time points and electrode sites, cluster and random permutation analyses were conducted (Maris and Oostenveld 2007) using the software package BESA Statistics v2.1 (BESA Software, Gräfelfing, Germany). Two separate ERP cluster analyses were calculated, one for P200 and one for LPC.

For the P200 cluster analysis, one-tailed-right (JOL minus no-JOL), paired *t*-tests were calculated for each time point (151) from 0 to 300 ms and electrode (40). For the LPC cluster analysis, one-tailed-right (JOL minus no-JOL), paired *t*-tests were calculated for each time point (551) from 300 to 1400 ms and electrode (40). For each cluster, only adjacent time points and contiguous electrode sites (with a maximum distance of 45 mm between neighboring sites, resulting in an average of 5.15 neighbors per electrode site) that fell below a *p*-value of 0.05 in the *t*-test were considered. The sum of *t*-values of a cluster’s single significant time points across electrodes was calculated as a test statistic. In random permutation analysis, 5000 random permutations were run in which the cluster *t* sum calculation was repeated for randomly shuffled datasets, in which the data were randomly reordered across conditions (JOL vs. no-JOL) and the cluster with the highest sum of *t*-values was kept. By these means, null distributions were created from the 5000 random permutation runs, and the critical *p*_rand_ values for the empirically derived ERP clusters were calculated.

### 2.7. Time Frequency Analyses

To analyze stimulus-induced power differences between the two conditions (JOL vs. no-JOL), the EEG data of the single trials (−1000 to 2000 ms around stimulus onset) were transformed into the time–frequency domain using a complex demodulation algorithm, which was implemented in BESA Research v7.1 (see Hoechstetter et al. 2004). The algorithm consists of a multiplication of the time domain signal with a complex periodic exponential function, having a frequency equal to the frequency under analysis, and subsequent low-pass filtering. The low-pass filter is a finite impulse response filter of Gaussian shape in the time domain, which is related to the envelope of the moving window in wavelet analysis. Time resolution was set to 78.8 ms (full power width at half maximum) and frequency resolution was set to 1.42 Hz (full power width at half maximum). Time–frequency data were exported in bins of 50 ms (from −1000 to 2000 ms around stimulus onset) and 1 Hz (from 2 to 30 Hz). Event-related power changes, time-locked to the onset of the task cue, were determined by calculating the temporal spectral evolution, i.e., power changes for all time–frequency points with power increases or decreases at time point (*t*) and frequency (*f*) related to mean power at frequency over a preceding baseline interval (Pfurtscheller and Aranibar 1977). The baseline interval was set from −300 to 0 ms before stimulus onset. The ERP was subtracted on each trial, separately for each condition, electrode, and participant (Kalcher and Pfurtscheller 1995). Percent power increase indicated ERS, whereas percent power decrease indicated ERD (Pfurtscheller and Lopes da Silva 1999).

Akin to ERP analysis, cluster and random permutation analysis was used to test the stimulus-induced power differences over multiple time–frequency points and electrode sites between conditions using the software package BESA Statistics v2.1 (BESA Software, Gräfelfing, Germany). In contrast to ERP analysis, the time–frequency data were analyzed with a two-step approach (Pastötter and Bäuml 2016; Tempel et al. 2020; Wirth et al. 2021). In the first step, a non-spatial cluster analysis was calculated, in which ERS/ERD spectrograms were averaged across the 40 electrodes and compared between conditions. In the second step, spatial topographies of clustered effects were identified.

Specifically, in the non-spatial cluster analysis, time–frequency data were averaged across all 40 electrodes and contrasted between conditions (JOL vs. no-JOL). For each time–frequency point from 0 to 1400 ms (29 time points) and from 2 to 30 Hz (29 frequency points), a one-tailed-left (JOL minus no-JOL), paired *t*-test was calculated. The sum of *t*-values of adjacent time–frequency points that fell below a *p*-value of 0.05 in the single *t*-tests was calculated as a test statistic. Random permutation analysis was calculated based on 5000 randomization runs. In each randomization run, time-frequency data of the two conditions (JOL, no-JOL) were interchanged randomly for each participant and *t*-tests were calculated for each time–frequency point. At the end of each run, *t*-values of adjacent time-frequency points that fell below a *p*-value of 0.05 were summed and the cluster with the highest sum of *t*-values was kept. By these means, a null distribution of cluster sums was created from the 5000 permutation runs, and the critical *p*_rand_ value for an empirically derived time–frequency cluster was estimated.

Empirical clusters with a *p*_rand_ value below 0.05 underwent spatial analysis. For each cluster, power changes were averaged across data points of the cluster’s maximum time range and maximum frequency range, separately for each electrode. One-tailed-left (JOL minus no-JOL), paired *t*-tests were calculated for all electrodes. Spatial topographies were identified by considering those electrodes that fell below a *p*-value of 0.05 in the *t*-test. No additional cluster analysis was calculated in order to avoid circular analysis.

### 2.8. Mediation Analyses

To investigate whether ERP components (e.g., P200 and LPC) mediate the JOL reactivity effect, multi-level mediating analyses were performed using the PROCESS function from the *bruceR* package in R (Bao 2023). In the mediation analyses, all numeric predictors were grand mean centered. The Monte Carlo method was used for sampling 1000 times, and the JOL condition was coded as 0, with the no-JOL condition coded as 1. The mediation analyses were calculated with the amplitude of the significant P200 or LPC cluster as a mediator variable, separately.

Additionally, two multi-level mediation analyses were calculated to investigate whether the time–frequency components (e.g., alpha and beta) mediate the JOL reactivity effect by using the PROCESS function in R. In the mediation analyses, the moderator variable was the power of alpha or beta frequency, separately.

## 3. Results

### 3.1. Behavioral Results

Bayesian analyses were performed to assess whether the documented findings favor the null (*H*_0_) or the alternative (*H*_1_) hypothesis. *BF*_10_ represents the strength of evidence favoring the alternative over the null hypothesis, with *BF*_10_ > 3 representing evidence supporting the alternative hypothesis over the null, and *BF*_10_ < 0.33 indicating evidence supporting the null hypothesis over the alternative (Mulder and Wagenmakers 2016). All Bayesian analyses presented below were conducted via JASP 0.12.2 (http://jasp-stats.org/, accessed on 27 September 2023).

**Figure 2:**
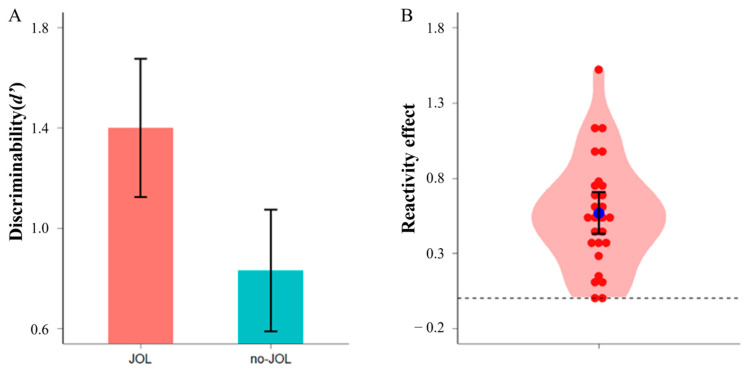
Panel (**A**): *d′* for JOL and no-JOL words. Panel (**B**): Violin plot depicting the distribution of the reactivity effect (i.e., the difference in *d′* between JOL and no-JOL conditions). Each red dot represents one participant’s reactivity effect score and the blue point represents group average. Error bars represent 95% CI.

Frequentist and Bayesian paired *t*-tests showed that *d′* for JOL words (*M* = 1.400, *SD* = 0.731) was significantly greater than that for no-JOL words (*M* = 0.832, *SD* = 0.644), difference = 0.568, 95% confidence interval (CI) [0.423, 0.714], *t*(26) = 8.031, *p* < 0.001, Cohen’s *d* = 1.546, *BF*_10_ = 2.475 × 10^6^ (see Figure 2). Twenty-five participants showed a positive reactivity effect, with the other two showing a null reactivity effect. Consistent with prior research (Li et al. 2022; Yang et al. 2015; Zechmeister and Shaughnessy 1980; Zhao et al. 2022), the current study successfully replicated the positive reactivity effect on word list learning.

### 3.2. ERP Results

#### 3.2.1. Results of Cluster Analyses

**Figure 3:**
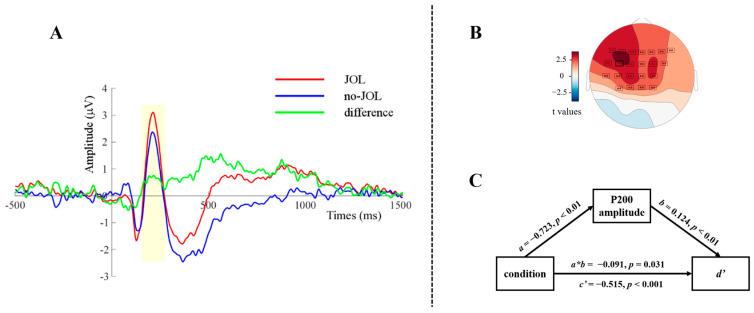
Panel (**A**) displays the average ERPs recorded at electrode points from the significant cluster in the P200 analysis. The time window of the significant cluster was from 159 to 250 ms (yellow shadow). The green line depicts the difference wave. In Panel (**B**), a topographic map illustrates the distribution of *t*-values and the P200 cluster’s significant electrodes. Panel (**C**) shows a schematic representation of the mediation analysis. In this model, the independent variable is condition (JOL vs. no-JOL), the mediating variable comprises the average ERPs of the P200 cluster’s significant electrodes, and the dependent variable is behavioral *d’*.

**Figure 4:**
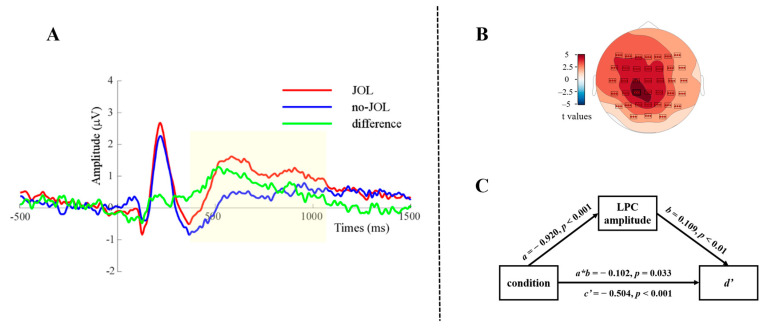
Panel (**A**) displays the average ERPs recorded at electrode points from the significant cluster in the LPC analysis. The time window of the significant cluster was from 348 ms to 1062 ms. The green line depicts the difference wave. In Panel (**B**), a topographic map illustrates the distribution of *t*-values and the LPC cluster’s significant electrodes. Panel (**C**) shows a schematic representation of the mediation analysis. In this model, the independent variable is condition (JOL vs. no-JOL), the mediating variable comprises the average ERPs of the LPC cluster’s significant electrodes, and the dependent variable is behavioral *d’*.

Cluster analyses were conducted to identify time windows of significant ERP effects related to the reactivity effect, separately for P200 and LPC. Both the P200 and the LPC analyses revealed a significant cluster for which the ERP amplitude under the JOL condition was significantly larger than the amplitude under the no-JOL condition (P200: *p*_rand_ = 0.003; LPC: *p*_rand_ = 0.003). For the P200 analysis, the cluster’s time window was from 159 to 250 ms after word onset (Figure 3A); for the LPC analysis, it was from 348 to 1062 ms after word onset (Figure 4A). These time windows were comparable to the ones reported in previous studies (Beato et al. 2012; Chen and Li 2013; Gan et al. 2016; Van Strien et al. 2007; Zhang et al. 2020). Regarding topographies, the P200 cluster was restricted to electrodes at left fronto-central sites (Figure 3B), whereas the LPC cluster was significant at all electrodes, with largest effect at left centro-parietal sites (Figure 4B). Peak electrodes were FC3 for the P200 cluster and CP1 for the LPC cluster.

#### 3.2.2. Results of Mediation Analyses

To investigate whether ERP components (P200 and LPC) mediate the JOL reactivity effect, two multi-level mediation analyses were performed using the PROCESS function from the *bruceR* package in R (Bao 2023). The first mediation analysis was calculated with the amplitude of the significant P200 cluster as the mediator variable (see Figure 3C). The results showed that condition significantly predicted the P200 amplitude (*a*), *β* = −0.723, *p* < 0.01, the P200 amplitude significantly predicted *d’* (*b*), *β* = 0.124, *p* < 0.01, the P200 amplitudes’ mediating effect (indirect effect, a*b) was significant, *β* = −0.091, *z* = −2.161, *p* = 0.031, MCMC 95% CI = [−0.187, −0.021], and the direct effect (*c’*) was significant, *β* = −0.515, *z* = −7.391, *p* < 0.001, MCMC 95% CI = [−0.651, −0.374]. These results indicated that the P200 amplitude partially mediates the JOL reactivity effect.

The second mediation analysis was calculated for the LPC cluster (see Figure 4C). The results showed that condition significantly predicted the LPC amplitude (*a*), *β* = −0.920, *p* < 0.001, the LPC amplitude significantly predicted *d*’ (*b*), *β* = 0.109, *p* < 0.01, the mediating effect of LPC amplitude (indirect effect, a*b) was significant, *β* = −0.102, *z* = −2.131, *p* = 0.033, MCMC 95% CI = [−0.207, −0.025], and the direct effect (*c’*) was also significant, *β* = −0.504, *z* = −6.625, *p* < 0.001, MCMC 95% CI = [−0.653, −0.350]. These results indicated that the LPC amplitude partially mediates the JOL reactivity effect.

### 3.3. Time-Frequency Results

#### 3.3.1. Results of Cluster Analyses

**Figure 5:**
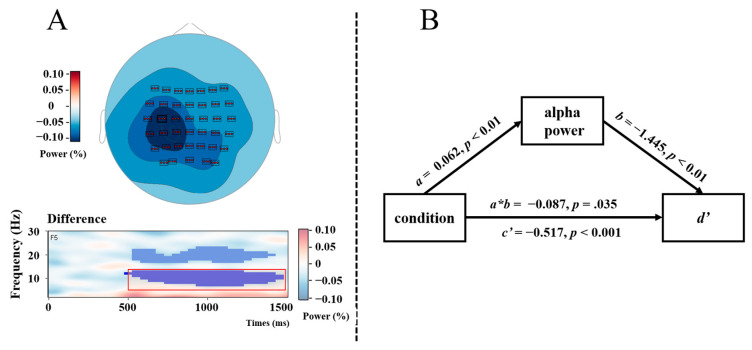
Panel (**A**) showcases two distinct elements. The upper portion features a topographic map that illustrates the alpha power differences between JOL and no-JOL conditions for the significant time–frequency range from 500 to 1450 ms and 7 to 13 Hz, as indicated by the non-spatial cluster analysis (NSA) of significant *t*-values shown below. Panel (**B**) depicts a schematic representation of the mediation analysis with alpha power as mediating variable, condition (JOL vs. no-JOL) as the independent variable, and *d’* as the dependent variable.

**Figure 6:**
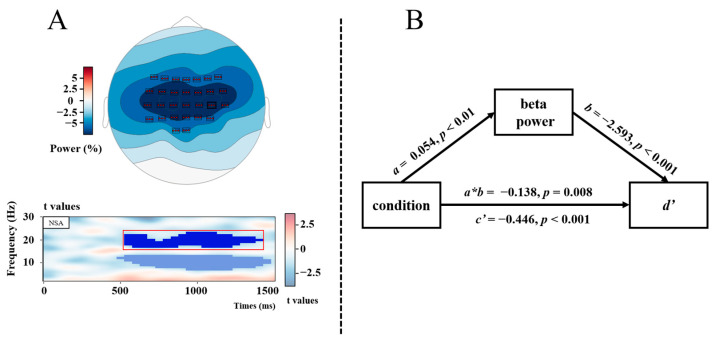
Panel (**A**) showcases two distinct elements. The upper portion features a topographic map that illustrates the beta power differences between JOL and no-JOL conditions for the significant time–frequency range from 550 to 1400 ms and 17 to 23 Hz, as indicated by the non-spatial cluster analysis (NSA) of significant *t*-values shown below. Panel (**B**) depicts a schematic representation of the mediation analysis with beta power as mediating variable, condition (JOL vs. no-JOL) as the independent variable, and *d’* as the dependent variable.

A non-spatial analysis revealed two clusters with stronger ERD in the JOL condition than in the no-JOL condition, with the first cluster in the alpha frequency ranging (7 Hz to 13 Hz) from 500 to 1450 ms (*p*_rand_ = 0.001), and the second cluster in the beta frequency ranging (17 Hz to 23 Hz) from 550 ms to 1400 ms (*p*_rand_ = 0.004). Spatial analyses revealed that the alpha power effect was significant at all electrodes with largest effect at left centro-parietal sites (Figure 5A), whereas the beta power effect was restricted to centro-parietal sites (Figure 6B). Peak electrodes were C3 for the alpha cluster and C4 for the beta cluster.

#### 3.3.2. Results of Mediation Analyses

Two multi-level mediation analyses were calculated to investigate whether time–frequency components (alpha and beta) mediate the JOL reactivity effect. The first analysis was calculated for alpha power (Figure 5B). The results showed that condition significantly predicted alpha power (*a*), *β* = 0.062, *p* < 0.01, alpha power significantly predicted *d’* (*b*), *β* = −1.445, *p* < 0.01, the mediating effect of alpha power (indirect effect, a*b) was significant, *β* = −0.087, *z* = −2.103, *p* = 0.035, MCMC 95% CI = [−0.169, −0.015], and the direct effect (*c’*) was also significant, *β* = −0.517, *z* = −7.056, *p* < 0.001, MCMC 95% CI = [−0.663, −0.374]. These results indicated that alpha power partially mediates the JOL reactivity effect.

The second mediation analysis was calculated for beta power (Figure 6B). The results showed that condition significantly predicted beta power (*a*), *β* = 0.054, *p* < 0.01, beta power significantly predicted *d’* (*b*), *β* = −2.593, *p* < 0.001, the mediating effect of beta power (indirect effect, a*b) was significant, *β* = −0.138, *z* = −2.655, *p* = 0.008, MCMC 95% CI = [−0.241, −0.044], and the direct effect (*c’*) was also significant, *β* = −0.466, *z* = −7.281, *p* < 0.001, MCMC 95% CI = [−0.592, −0.342]. These results suggested that, just like alpha power, beta power also partially mediates the JOL reactivity effect.

## 4. Discussion

Although a set of recent studies consistently demonstrated that making concurrent JOLs can reactively change memory itself (Janes et al. 2018; Li et al. 2022, 2023; Mitchum et al. 2016; Myers et al. 2020; Rivers et al. 2021; Soderstrom et al. 2015; Tekin and Roediger 2020; Witherby and Tauber 2017; Zhao et al. 2022), no research has been conducted to explore the neurocognitive underpinnings associated with the effect. The current study is the first to explore neural features associated with the effect. The behavioral results successfully replicated the positive reactivity effect on word list learning (Li et al. 2022, 2023; Shi et al. 2023; Yang et al. 2015; Zechmeister and Shaughnessy 1980; Zhao et al. 2022). More importantly, the EEG results demonstrated that making JOLs increased the ERP amplitudes of P200 and LPC and decreased stimulus-induced alpha and beta power (larger ERDs). Furthermore, both ERPs (P200 and LPC) and ERDs (alpha and beta) partially mediated the positive reactivity effect observed here.

Numerous studies have found that increased P200 amplitude is related to enhanced attention during encoding (Carreiras et al. 2005; Gan et al. 2016; Kanske et al. 2011; Leuthold et al. 2015; Lu et al. 2010; Missonnier et al. 2007). The present study found that making JOLs increased the P200 amplitude, which supports the main proposal of the enhanced learning engagement theory that when asked to make JOLs, participants need to look for some cues to inform JOL formation, and thus making JOLs increases the level of attentional processing of study items. Furthermore, the present study also found that making JOLs increased desynchronization in the alpha band. Pastötter et al. (2011) claimed that alpha desynchronization reflects an increase in attentional engagement during a learning task. Previous EEG-fMRI research found that alpha oscillations are linked to regulation of the default mode network, which is a task-negative network (Bowman et al. 2017; Clancy et al. 2022; Jann et al. 2009; Knyazev et al. 2011). When a given task requires more resources, this network exhibits greater deactivation (Bowman et al. 2017; Jann et al. 2009; Knyazev et al. 2011). The finding that making JOLs reduced alpha power suggests that the requirement of making JOLs may increase attentional engagement in the learning process, which in turn produces a positive reactivity effect. The mediation results supported this assumption.

As elaborated above, the positive reactivity effect can also be explained by the elaborative processing theory, which claims that making JOLs produces the positive reactivity effect by inducing more elaborative processing of study items (Mitchum et al. 2016; Sahakyan et al. 2004; Tekin and Roediger 2020). Going beyond previous behavioral research (Rivers et al. 2021; Soderstrom et al. 2015), the current study provides neurocognitive evidence that making JOLs increased the amplitude of LPC and reduced beta power during the encoding phase. These results provide objective evidence for the elaboration processing explanation (Mitchum et al. 2016; Sahakyan et al. 2004; Tekin and Roediger 2020). Specifically, prior studies have established that LPC amplitude is positively related to elaborative processing (Chen and Li 2013; De Grauwe et al. 2010; Zhang et al. 2020), and beta ERD reflects an increase in (semantic) elaboration and deep encoding (Guran et al. 2019; Hanslmayr et al. 2008; Klimesch et al. 1997; Pastötter and Bäuml 2016). The current study showed that making JOLs increased the LPC amplitude and beta ERD. In addition, LPC amplitude and beta power partially mediated the positive reactivity effect. These results indicated that the requirement of making JOLs enhances memory performance partially through inducing deep and elaborative encoding.

Overall, the current study revealed that the amplitude of LPC as well as the ERD of alpha and beta bands partially mediate the positive reactivity effect. It is worth noting that the time courses of the LPC amplitude (348–1062 ms), alpha (500–1450 ms), and beta (550–1400 ms) bands were different. Such evidence indicates that the mechanisms proposed by the enhanced learning engagement theory and those proposed by the elaborative processing theory are not mutually exclusive, and these mechanisms (i.e., enhanced attentional and elaborative processing) may jointly contribute to the JOL reactivity effect. Specifically, to make a JOL, participants need to look for some cues to inform JOL formation (e.g., Koriat 1997; Rhodes 2016; Yang et al. 2017). During the process of word encoding, making JOLs increases the metacognitive monitoring process (Lei et al. 2020) and thus enhances learning engagement (Shi et al. 2023). Additionally, searching for cues to inform JOL formation also enhances item-specific processing (Chang and Brainerd 2024; Senkova and Otani 2021; Zhao et al. 2023a, 2023b), thus producing more elaborative processing. Cognitive neural activities related to learning engagement (alpha ERD) and those related to elaborative encoding (LPC amplitude and beta ERD) together contribute to the generation of the positive reactivity effect.

## 5. Limitations

As far as we know, the present study is the first to explore neurocognitive features associated with the reactivity effect. Our results preliminarily revealed the neurocognitive features of the reactivity effect. However, the current study did not provide direct evidence to justify the causal roles of enhanced learning engagement and elaborative processing in the reactivity effect, even though the mediation results are statistically significant. Future research is encouraged to further explore their potential causal roles through non-invasive electrical or magnetic stimulation. In addition, future research should determine the contribution of attentional and elaborative processing to the reactivity effect in different patient groups with specific deficits in attention (e.g., ADHD) or deficits in elaborative encoding (e.g., semantic dementia).

The current study used word lists as study stimuli. Many other studies employed related word pairs as stimuli to investigate the reactivity effect (Double et al. 2018; Myers et al. 2020; Rivers et al. 2021; Witherby and Tauber 2017). The cognitive underpinnings of the reactivity effects on learning of word lists and word pairs tend to be different (Li et al. 2022; Rivers et al. 2021; Soderstrom et al. 2015; Zhao et al. 2022). Thus, future research is needed to further explore the neurocognitive features of the reactivity effect on memory for related word pairs and other learning materials.

In the current study, a between-list design was employed, in which JOL and no-JOL words were presented in a blocked manner (i.e., presented in different study lists). As the experimental operations were consistent across items in each list, participants might have expectations about metacognitive monitoring in the JOL lists. This could induce changes in cognitive neural signals during the encoding phase. Future research is encouraged to test the replicability of the present findings using a within-list design, in which JOL and no-JOL words are presented in a randomly interleaved order (e.g., a JOL word, a no-JOL word, a no-JOL word, a JOL word…). Such a within-list design is expected to diminish the confounding effect of expectation.

## 6. Conclusions

Making JOLs increases P200 and LPC amplitudes and decreases alpha and beta power during the encoding phase. The reactivity effect is partially mediated by changes in these neurocognitive signals. The enhanced learning engagement and elaborative processing theories are viable explanations for the reactivity effect on word list learning.

## Data Availability

The data contained in this project are publicly available at Open Science Framework (https://osf.io/8utmf/, accessed on 27 September 2023).

## Note

1 In the current study, *d*′ was calculated as the signed difference between Z-transformed hit rate and Z-transformed false alarm rate (Banks 1970; Stanislaw and Todorov 1999).

## Appendix A

Participants successfully provided item-by-item JOLs to 99.0% (*SD* = 1.0%) of the words in the JOL lists. The average of JOLs was 5.125 (*SD* = 0.922). For each participant, a Gamma (*G*) correlation was calculated to measure the relative accuracy of JOLs. The averaged *G* across participants was 0.121 (*SD* = 0.270, 95% CI [0.014, 0.228]), which was significantly greater than the chance level (i.e., 0), *t*(26) = 2.321, *p* = 0.028, Cohen’s *d* = 0.447, indicating that participants were somewhat accurate in monitoring their learning of words.

## References

[B1-jintelligence-12-00044] Banks William P. (1970). Signal detection theory and human memory. Psychological Bulletin.

[B2-jintelligence-12-00044] Bao Han-Wu-Shuang (2023). Broadly Useful Convenient and Efficient R Functions (Version 2023.9). https://CRAN.R-project.org/package=bruceR.

[B3-jintelligence-12-00044] Beato Maria Soledad, Boldini Angela, Cadavid Sadavid (2012). False memory and level of processing effect: An event-related potential study. Neuroreport.

[B4-jintelligence-12-00044] Bowman Anthony D., Griffis Joseph C., Visscher Kristina M., Dobbins Allan C., Gawne Timothy J., DiFrancesco Mark W., Szaflarski Jerzy P. (2017). Relationship between alpha rhythm and the default mode network: An EEG-fMRI study. Journal of Clinical Neurophysiology.

[B5-jintelligence-12-00044] Cai Qing, Brysbaert Marc (2010). SUBTLEX-CH: Chinese word and character frequencies based on film subtitles. PLoS ONE.

[B6-jintelligence-12-00044] Carreiras Manuel, Vergara Marta, Barber Horacio (2005). Early event-related potential effects of syllabic processing during visual word recognition. Journal of Cognitive Neuroscience.

[B7-jintelligence-12-00044] Chang Mingyu, Brainerd Charles (2024). Judgments of learning reactivity on item-specific and relational processing. Journal of Intelligence.

[B8-jintelligence-12-00044] Chen Lingli, Li Ling (2013). Context modulates neural activity of N400 and P600 to visual words. Journal of Neurolinguistics.

[B9-jintelligence-12-00044] Clancy Kevin J., Andrzejewski Jeremy A., You Yuqi, Rosenberg Jens T., Ding Mingzhou, Li Wen (2022). Transcranial stimulation of alpha oscillations up-regulates the default mode network. Proceedings of the National Academy of Sciences of the United States of America.

[B10-jintelligence-12-00044] De Grauwe Sophie, Swain Abigail, Holcomb Phillip J., Ditman Tali, Kuperberg Gina R. (2010). Electrophysiological insights into the processing of nominal metaphors. Neuropsychologia.

[B11-jintelligence-12-00044] Delorme Arnaud, Makeig Scott (2004). EEGLAB: An open source toolbox for analysis of single-trial EEG dynamics including independent component analysis. Journal of Neuroscience Methods.

[B12-jintelligence-12-00044] Double Kit S., Birney Damian P. (2019). Reactivity to measures of metacognition. Frontiers in Psychology.

[B13-jintelligence-12-00044] Double Kit S., Birney Damian P., Walker Sarah A. (2018). A meta-analysis and systematic review of reactivity to judgements of learning. Memory.

[B14-jintelligence-12-00044] Dunlosky John, Hertzog Christopher (1997). Older and younger adults use a functionally identical algorithm to select items for restudy during multitrial learning. The Journals of Gerontology Series B: Psychological Sciences and Social Sciences.

[B15-jintelligence-12-00044] Dunlosky John, Thiede Keith W. (2004). Causes and constraints of the shift-to-easier-materials effect in the control of study. Memory & Cognition.

[B16-jintelligence-12-00044] Einstein Gillies O., McDaniel Mark A. (1997). Aging and mind wandering: Reduced inhibition in older adults?. Experimental Aging Research.

[B17-jintelligence-12-00044] Faul Franz, Erdfelder Edgar, Lang Albert-Georg, Buchner Axel (2007). G*Power 3: A flexible statistical power analysis program for the social, behavioral, and biomedical sciences. Behavior Research Methods.

[B18-jintelligence-12-00044] Finn Bridgid (2008). Framing effects on metacognitive monitoring and control. Memory & Cognition.

[B19-jintelligence-12-00044] Fortin Julie, Grondin Simon, Blanchet Sophie (2021). Event-related potentials of episodic encoding after traumatic brain injury in older adults. Brain Research.

[B20-jintelligence-12-00044] Gan Tian, Fang Wei, Ge Liezhong (2016). Colours’ impact on morality: Evidence from event-related potentials. Scientific Reports.

[B21-jintelligence-12-00044] Guran Catherine-Noémie Alexandrina, Herweg Nora Alicia, Bunzeck Nico (2019). Age-related decreases in the retrieval practice effect directly relate to changes in alpha-beta oscillations. The Journal of Neuroscience.

[B22-jintelligence-12-00044] Hanslmayr Simon, Spitzer Bernhard, Bäuml Karl-Heinz (2008). Brain oscillations dissociate between semantic and nonsemantic encoding of episodic memories. Cerebral Cortex.

[B23-jintelligence-12-00044] Hanslmayr Simon, Volberg Gregor, Wimber Maria, Raabe Makus, Greenlee Mark W., Bäuml Karl-Heinz T. (2011). The relationship between brain oscillations and BOLD signal during memory formation: A combined EEG-fMRI study. The Journal of Neuroscience.

[B24-jintelligence-12-00044] Hoechstetter Karsten, Bornfleth Harald, Weckesser Dieter, Ille Nicole, Berg Patrick, Scherg Michael (2004). BESA source coherence: A new method to study cortical oscillatory coupling. Brain Topography.

[B25-jintelligence-12-00044] Janes Jessica L., Rivers Michelle L., Dunlosky John (2018). The influence of making judgments of learning on memory performance: Positive, negative, or both?. Psychonomic Bulletin & Review.

[B26-jintelligence-12-00044] Jann Kay B., Dierks Thomas, Boesch Chris, Kottlow Mara, Strik Werner, Koenig Toenig (2009). BOLD correlates of EEG alpha phase-locking and the fMRI default mode network. NeuroImage.

[B27-jintelligence-12-00044] Jia Xi, Gao Chuanji, Li Baoming, Shinkareva Svetlana V., Guo Chunyan (2021). Effects of retrieval and emotion on within-item associative memory—Evidence from ERP and oscillatory subsequent memory effects. Biological Psychology.

[B28-jintelligence-12-00044] Jordão Magda, Ferreira-Santos Fernando, Pinho Maria Salomé, Jacques Peggy L. St. (2019). Meta-analysis of aging effects in mind wandering: Methodological and sociodemographic factors. Psychology and Aging.

[B29-jintelligence-12-00044] Jung Tzyy-Ping, Humphries Colin, Lee Te-Won, Makeig Scott, McKeown Matin J., Iragui Vicente, Sejnowski Terrence J. (1998). Extended ICA removes artifacts from electroencephalographic recordings. Paper presented at the Advances in Neural Information Processing Systems.

[B30-jintelligence-12-00044] Kalcher Joachim, Pfurtscheller Gert (1995). Discrimination between phase-locked and non-phase-locked event-related EEG activity. Electroencephalography and Clinical Neurophysiology.

[B31-jintelligence-12-00044] Kanske Philipp, Plitschka Jan, Kotz Sonja A. (2011). Attentional orienting towards emotion: P2 and N400 ERP effects. Neuropsychologia.

[B32-jintelligence-12-00044] Katerman Brandon S., Li Yuxuan, Pazdera Jessie K., Keane Connor, Kahana Michael J. (2021). EEG biomarkers of free recall. NeuroImage.

[B33-jintelligence-12-00044] Kim Alice S. N., Wiseheart Melody, Wong-Kee-You Audrey M. B., Le B. T., Moreno Sylvain, Rosenbaum Shayna (2020). Specifying the neural basis of the spacing effect with multivariate ERP. Neuropsychologia.

[B34-jintelligence-12-00044] Kleiner Mario, Brainard David, Pelli Denis (2007). What’s new in Psychtoolbox-3? Paper presented at the Perception 36 ECVP Abstract Supplement, Arezzo, Italy, August 27–31.

[B35-jintelligence-12-00044] Klimesch Wolfgang, Doppelmayr Michael, Pachinger Thomas, Russegger Harald (1997). Event-related desynchronization in the alpha band and the processing of semantic information. Cognitive Brain Research.

[B36-jintelligence-12-00044] Knyazev Gennady G., Slobodskoj-Plusnin Jaroslav Y., Bocharov Andrey V., Pylkova Liudmila V. (2011). The default mode network and EEG alpha oscillations: An independent component analysis. Brain Research.

[B37-jintelligence-12-00044] Koriat Asher (1997). Monitoring one’s own knowledge during study: A cue-utilization approach to judgments of learning. Journal of Experimental Psychology: General.

[B38-jintelligence-12-00044] Krawietz Sabine A., Tamplin Andrea K., Radvansky Gabriel A. (2012). Aging and mind wandering during text comprehension. Psychology and Aging.

[B39-jintelligence-12-00044] Kubik Veit, Koslowski Kenneth, Schubert Torsten, Aslan Alp (2022). Metacognitive judgments can potentiate new learning: The role of covert retrieval. Metacognition and Learning.

[B40-jintelligence-12-00044] Lei Wei, Chen Jing, Yang Chunliang, Guo Yiqun, Feng Pan, Feng Tingyong, Li Hong (2020). Metacognition-related regions modulate the reactivity effect of confidence ratings on perceptual decision-making. Neuropsychologia.

[B41-jintelligence-12-00044] Leuthold Hartmut, Kunkel Angelika, Mackenzie Ian G., Filik Ruth (2015). Online processing of moral transgressions: ERP evidence for spontaneous evaluation. Social Cognitive Affective Neuroscience.

[B42-jintelligence-12-00044] Li Baike, Shanks David R., Zhao Wenbo, Hu Xiao, Luo Liang, Yang Chunliang (2024). Do changed learning goals explain why metamemory judgments reactively affect memory?. Journal of Memory and Language.

[B43-jintelligence-12-00044] Li Baike, Zhao Wenbo, Shi Aaike, Zhong Yongen, Hu Xiao, Liu Meng, Luo Liang, Yang Chunliang (2023). Does the reactivity effect of judgments of learning transfer to learning of new information?. Memory.

[B44-jintelligence-12-00044] Li Baike, Zhao Wenbo, Zheng Jun, Hu Xiao, Su Ning, Fan Tian, Yin Yue, Liu Meng, Yang Chunliang, Luo Liang (2022). Soliciting judgments of forgetting reactively enhances memory as well as making judgments of learning: Empirical and meta-analytic tests. Memory & Cognition.

[B45-jintelligence-12-00044] Lu Aitao, Xu Guiping, Jin Hua, Mo Lei, Zhang Jijia, Zhang John X. (2010). Electrophysiological evidence for effects of color knowledge in object recognition. Neuroscience Letters.

[B46-jintelligence-12-00044] Maris Eric, Oostenveld Robert (2007). Nonparametric statistical testing of EEG- and MEG-data. Journal of Neuroscience Methods.

[B47-jintelligence-12-00044] Metcalfe Janet, Finn Bridgid (2013). Metacognition and control of study choice in children. Metacognition and Learning.

[B48-jintelligence-12-00044] Missonnier Pascal, Deiber M.-P., Gold Gabriel, Herrmann Francois R., Millet Philippe, Michon Agnès, Fazio-Costa Lara, Ibañez Vicente, Giannakopoulos Panteleimon (2007). Working memory load–related electroencephalographic parameters can differentiate progressive from stable mild cognitive impairment. Neuroscience.

[B49-jintelligence-12-00044] Mitchum Ainsley L., Kelley Colleen M., Fox Mark C. (2016). When asking the question changes the ultimate answer: Metamemory judgments change memory. Journal of Experimental Psychology: General.

[B50-jintelligence-12-00044] Mulder Joris, Wagenmakers Eric-Jan (2016). Editors’ introduction to the special issue “Bayes factors for testing hypotheses in psychological research: Practical relevance and new developments”. Journal of Mathematical Psychology.

[B51-jintelligence-12-00044] Myers Sarah J., Rhodes Matthew G., Hausman Hannah E. (2020). Judgments of learning (JOLs) selectively improve memory depending on the type of test. Memory & Cognition.

[B52-jintelligence-12-00044] Nelson Thornas O., Narens Louris, Metcalfe Janet, Shimamura Arthur P. (1994). Why investigate metacognition. Metacognition: Knowing about Knowing.

[B53-jintelligence-12-00044] Packard Pau A., Steiger Tineke K., Fuentemilla Lluís, Bunzeck Nico (2020). Neural oscillations and event-related potentials reveal how semantic congruence drives long-term memory in both young and older humans. Scientific Reports.

[B54-jintelligence-12-00044] Pastötter Bernhard, Bäuml Karl-Heinz T. (2016). Reversing the testing effect by feedback: Behavioral and electrophysiological evidence. Cognitive, Affective, & Behavioral Neuroscience.

[B55-jintelligence-12-00044] Pastötter Bernhard, Bäuml Karl-Heinz T., Hanslmayr Simon (2008). Oscillatory brain activity before and after an internal context change—Evidence for a reset of encoding processes. NeuroImage.

[B56-jintelligence-12-00044] Pastötter Bernhard, Schicker Sabine, Niedernhuber Julia, Bäuml Karl-Heinz T. (2011). Retrieval during learning facilitates subsequent memory encoding. Journal of Experimental Psychology: Learning, Memory, and Cognition.

[B57-jintelligence-12-00044] Pfurtscheller Gert (1992). Event-related synchronization (ERS): An electrophysiological correlate of cortical areas at rest. Electroencephalography and Clinical Neurophysiology.

[B58-jintelligence-12-00044] Pfurtscheller Gert, Aranibar A. (1977). Event-related cortical desynchronization detected by power measurements of scalp EEG. Electroencephalography and Clinical Neurophysiology.

[B59-jintelligence-12-00044] Pfurtscheller Gert, Lopes da Silva Fernando H. (1999). Event-related EEG/MEG synchronization and desynchronization: Basic principles. Clinical Neurophysiology.

[B60-jintelligence-12-00044] Rhodes Matthew G. (2016). Judgments of learning: Methods, data, and theory. The Oxford Handbook of Metamemory.

[B61-jintelligence-12-00044] Rhodes Matthew G., Castel Alan D. (2009). Metacognitive illusions for auditory information: Effects on monitoring and control. Psychonomic Bulletin & Review.

[B62-jintelligence-12-00044] Rivers Michelle L., Janes Jessica L., Dunlosky John (2021). Investigating memory reactivity with a within-participant manipulation of judgments of learning: Support for the cue-strengthening hypothesis. Memory.

[B63-jintelligence-12-00044] Sahakyan Lili, Delaney Peter F., Kelley Colleen M. (2004). Self-evaluation as a moderating factor of strategy change in directed forgetting benefits. Psychonomic Bulletin & Review.

[B64-jintelligence-12-00044] Sanquist Thomas F., Rohrbaugh John W., Syndulko Karl, Lindsley Donald B. (1980). Electrocortical signs of levels of processing: Perceptual analysis and recognition memory. Psychophysiology.

[B65-jintelligence-12-00044] Seli Paul, Risko Evan F., Smilek Daniel, Schacter Daniel L. (2016). Mind-wandering with and without intention. Trends in Cognitive Sciences.

[B66-jintelligence-12-00044] Senkova Olesya, Otani Hajima (2021). Making judgments of learning enhances memory by inducing item-specific processing. Memory & Cognition.

[B67-jintelligence-12-00044] Shi Aaike, Xu Chenyuqi, Zhao Wenbo, Shanks David R., Hu Xiao, Luo Liang, Yang Chunliang (2023). Judgments of learning reactively facilitate visual memory by enhancing learning engagement. Psychonomic Bulletin & Review.

[B68-jintelligence-12-00044] Soderstrom Nicholas C., Clark Colin T., Halamish Vered, Bjork Elizabeth Ligon (2015). Judgments of learning as memory modifiers. Journal of Experimental Psychology: Learning, Memory, and Cognition.

[B69-jintelligence-12-00044] Stanislaw Harold, Todorov Natasha (1999). Calculation of signal detection theory measures. Behavior Research Methods, Instruments, & Computers.

[B70-jintelligence-12-00044] Tallon-Baudry Catherine, Bertrand Olivier (1999). Oscillatory gamma activity in humans and its role in object representation. Trends in Cognitive Sciences.

[B71-jintelligence-12-00044] Tauber Sarah K., Witherby Amber E. (2019). Do judgments of learning modify older adults’ actual learning?. Psychology and Aging.

[B72-jintelligence-12-00044] Tekin Eylul, Roediger Henry L. (2020). Reactivity of judgments of learning in a levels-of-processing paradigm. Zeitschrift für Psychologie.

[B73-jintelligence-12-00044] Tempel Tobias, Frings Christian, Pastötter Bernhard (2020). EEG beta power increase indicates inhibition in motor memory. International Journal of Psychophysiology.

[B74-jintelligence-12-00044] Thiede Keith W. (1999). The importance of monitoring and self-regulation during multitrial learning. Psychonomic Bulletin & Review.

[B75-jintelligence-12-00044] Thiede Keith W., Dunlosky John (1999). Toward a general model of self-regulated study: An analysis of selection of items for study and self-paced study time. Journal of Experimental Psychology: Learning, Memory, and Cognition.

[B76-jintelligence-12-00044] Van Strien Jan W., Verkoeijen Peter P. J. L., Van der Meer Nelly, Franken Ingmar H. A. (2007). Electrophysiological correlates of word repetition spacing: ERP and induced band power old/new effects with massed and spaced repetitions. International Journal of Psychophysiology.

[B77-jintelligence-12-00044] Verkoeijen Peter P. J. L., Rikers Remy M. J. P., Schmidt Henk G. (2005). The effects of prior knowledge on study-time allocation and free recall: Investigating the discrepancy reduction model. The Journal of Psychology.

[B78-jintelligence-12-00044] Winograd Eugene, Vom Saal Walter (1966). Discriminability of association value in recognition memory. Journal of Experimental Psychology.

[B79-jintelligence-12-00044] Wirth Michael, Pastötter Bernhard, Bäuml Karl-Heinz T. (2021). Oscillatory correlates of selective restudy. Frontiers in Human Neuroscience.

[B80-jintelligence-12-00044] Witherby Amber E., Tauber Sarah K. (2017). The influence of judgments of learning on long-term learning and short-term performance. Journal of Applied Research in Memory and Cognition.

[B81-jintelligence-12-00044] Yang Chunliang, Potts Rosalind, Shanks David R. (2017). Metacognitive unawareness of the errorful generation benefit and Its effects on self-regulated learning. Journal of Experimental Psychology: Learning, Memory, and Cognition.

[B82-jintelligence-12-00044] Yang Haiyan, Cai Ying, Liu Qi, Zhao Xiao, Wang Qiang, Chen Chuansheng, Xue Gui (2015). Differential neural correlates underlie judgment of learning and subsequent memory performance. Frontiers in Psychology.

[B83-jintelligence-12-00044] Zechmeister Eugene B., Shaughnessy John J. (1980). When you know that you know and when you think that you know but you don’t. Bulletin of the Psychonomic Society.

[B84-jintelligence-12-00044] Zhang Jie, Li Xiaohua, Guo Chunyan (2020). The neurocognitive features in survival processing: An ERP study. International Journal of Psychophysiology.

[B85-jintelligence-12-00044] Zhao Wanlin, Li Baike, Shanks David R., Zhao Wenbo, Zheng Jun, Hu Xiao, Su Ningxin, Fan Tian, Yin Yue, Luo Liang (2022). When judging what you know changes what you really know: Soliciting metamemory judgments reactively enhances children’s learning. Child Development.

[B86-jintelligence-12-00044] Zhao Wenbo, Li Jiaojiao, Shanks David R., Li Baike, Hu Xiao, Yang Chuanlaing, Luo Liang (2023a). Metamemory judgments have dissociable reactivity effects on item and interitem relational memory. Journal of Experimental Psychology: Learning, Memory, and Cognition.

[B87-jintelligence-12-00044] Zhao Wenbo, Yin Yue, Hu Xiao, Shanks David R., Yang Chunliang, Luo Liang (2023b). Memory for inter-item relations is reactively disrupted by metamemory judgments. Metacognition and Learning.

